# Supplementary material for: Effects of starch sugar by-product on rumen in vitro digestibility, in situ disappearance rate, and milking productivity of the lactating dairy cow
Source: PeerJ. 2022 Feb 22;10:e12998. doi: 10.7717/peerj.12998 (PMC8877396; doi:10.7717/peerj.12998)
Supplement: Supplemental Information 1 [file peerj-10-12998-s001.docx]

Table A1. Fatty acid composition of starch sugar by-product

| Fatty acid (%) | Chemical  formula | Mean | Median | SD | MIN | MAX | Skewness^1^ | 2×SE_S_^2^ | Kurtosis^3^ | 2×SE_k_^4^ |
| --- | --- | --- | --- | --- | --- | --- | --- | --- | --- | --- |
| Caprylic acid | C_8:0_ | 0.02 | 0.01 | 0.01 | 0.01 | 0.04 | 0.86 | 1.55 | -0.91 | 3.10 |
| Lauric acid | C_12:0_ | 0.02 | 0.01 | 0.02 | 0.01 | 0.05 | 1.03 | 1.55 | -0.78 | 3.10 |
| Myristic acid | C_14:0_ | 0.09 | 0.08 | 0.02 | 0.05 | 0.11 | -0.65 | 1.55 | 1.54 | 3.10 |
| Palmitic acid | C_16:0_ | 40.11 | 40.24 | 5.85 | 30.52 | 47.28 | -0.44 | 1.55 | -1.37 | 3.10 |
| Margaric acid | C_17:0_ | 0.19 | 0.15 | 0.08 | 0.06 | 0.30 | -0.16 | 1.55 | 1.45 | 3.10 |
| Stearic acid | C_18:0_ | 3.24 | 3.13 | 0.65 | 2.24 | 4.23 | 0.06 | 1.55 | -0.86 | 3.10 |
| Arachidic acid | C_20:0_ | 0.29 | 0.27 | 0.06 | 0.19 | 0.37 | 0.05 | 1.55 | -0.67 | 3.10 |
| Behenic acid | C_22:0_ | 0.11 | 0.08 | 0.07 | 0.03 | 0.24 | 1.21 | 1.55 | 0.54 | 3.10 |
| Palmitoleic acid | C_16:1_ | 0.17 | 0.14 | 0.07 | 0.09 | 0.28 | 0.86 | 1.55 | -1.17 | 3.10 |
| Magaoleic acid | C_17:1_ | 0.11 | 0.11 | 0.06 | 0.04 | 0.22 | 0.29 | 1.55 | -1.04 | 3.10 |
| Oleic acid | C_18:1n9_ | 14.01 | 13.96 | 1.43 | 11.9 | 16.62 | 0.22 | 1.55 | 0.07 | 3.10 |
| Linoleic acid | C_18:2n6_ | 36.77 | 33.45 | 7.10 | 29.1 | 49.26 | 0.53 | 1.55 | -1.12 | 3.10 |
| Linolenic acid | C_18:3n3_ | 1.87 | 1.39 | 0.86 | 0.96 | 3.33 | 0.67 | 1.55 | -1.10 | 3.10 |
| Arachidonic acid | C_20:4n6_ | 0.25 | 0.28 | 0.11 | 0.05 | 0.37 | -0.79 | 1.55 | -0.76 | 3.10 |

DM, dry matter; CP, crude protein; EE, ether extract; NDF, neutral detergent fiber; ADF, acid detergent fiber; WSC, water soluble carbohydrate; GE, gross energy; SD, Standard deviation; MIN, Minimum value in database, MAX, Maximum value in database.

^1^The degree of asymmetry of a distribution around its mean where 0 ± 2 × Ses = normal.

^2^SEs, square root (6/n).

^3^Characterizes the relative peakedness or flatness of a distribution, where 0 ± 2 × Sek = normal.

^4^SEk, square root (24/n)
