# Supplementary material for: Effects of starch sugar by-product on rumen in vitro digestibility, in situ disappearance rate, and milking productivity of the lactating dairy cow
Source: PeerJ. 2022 Feb 22;10:e12998. doi: 10.7717/peerj.12998 (PMC8877396; doi:10.7717/peerj.12998)
Supplement: Supplemental Information 2 [file peerj-10-12998-s002.docx]

Table A2. Amino acid content of starch sugar by-product

| Amino acid (mg/g) | Mean | Median | SD | MIN | MAX | Skewness^1^ | 2×SE_S_^2^ | Kurtosis^3^ | 2×SE_k_^4^ |
| --- | --- | --- | --- | --- | --- | --- | --- | --- | --- |
| Aspartic acid | 9.47 | 7.85 | 4.10 | 4.29 | 19.55 | 1.47 | 1.55 | 2.74 | 3.10 |
| Glutamic acid | 23.90 | 17.44 | 14.61 | 10.35 | 64.43 | 2.37 | 1.55 | 6.34 | 3.10 |
| Serine | 5.95 | 4.65 | 3.24 | 2.59 | 14.76 | 2.18 | 1.55 | 5.56 | 3.10 |
| Histidine | 2.31 | 1.67 | 1.14 | 1.01 | 5.17 | 1.63 | 1.55 | 3.10 | 3.10 |
| Glycine | 4.59 | 3.81 | 1.80 | 2.09 | 8.70 | 1.09 | 1.55 | 1.42 | 3.10 |
| Threonine | 4.62 | 3.91 | 2.05 | 2.18 | 9.89 | 1.74 | 1.55 | 3.86 | 3.10 |
| Arginine | 5.05 | 4.25 | 1.73 | 2.46 | 8.65 | 0.74 | 1.55 | 0.26 | 3.10 |
| Alanine | 9.59 | 7.43 | 5.44 | 4.09 | 24.44 | 2.22 | 1.55 | 5.72 | 3.10 |
| Tyrosine | 4.64 | 3.53 | 2.67 | 1.94 | 11.78 | 2.09 | 1.55 | 5.04 | 3.10 |
| Valine | 6.68 | 4.98 | 3.03 | 3.06 | 14.08 | 1.49 | 1.55 | 2.53 | 3.10 |
| Methionine | 2.88 | 2.11 | 1.64 | 1.07 | 7.04 | 1.74 | 1.55 | 3.51 | 3.10 |
| Phenylalanine | 6.79 | 5.32 | 3.78 | 2.95 | 17.01 | 2.16 | 1.55 | 5.44 | 3.10 |
| Isoleucine | 4.87 | 3.53 | 2.51 | 2.13 | 11.2 | 1.77 | 1.55 | 3.63 | 3.10 |
| Leucine | 15.21 | 11.11 | 9.61 | 6.48 | 42.01 | 2.41 | 1.55 | 6.53 | 3.10 |
| Lysine | 2.74 | 2.00 | 1.04 | 1.29 | 4.35 | 1.78 | 1.55 | -1.73 | 3.10 |
| Proline | 9.71 | 7.22 | 5.46 | 4.40 | 24.80 | 2.33 | 1.55 | 6.21 | 3.10 |

DM, dry matter; CP, crude protein; EE, ether extract; NDF, neutral detergent fiber; ADF, acid detergent fiber; WSC, water soluble carbohydrate; GE, gross energy; SD, Standard deviation; MIN, Minimum value in database, MAX, Maximum value in database.

^1^The degree of asymmetry of a distribution around its mean where 0 ± 2 × Ses = normal.

^2^SEs, square root (6/n).

^3^Characterizes the relative peakedness or flatness of a distribution, where 0 ± 2 × Sek = normal.

^4^SEk, square root (24/n).
